# Effects of Dietary Supplementation with Vitamin A on Antioxidant and Intestinal Barrier Function of Broilers Co-Infected with Coccidia and *Clostridium perfringens*

**DOI:** 10.3390/ani12233431

**Published:** 2022-12-05

**Authors:** Peng Li, Chengao Liu, Junlong Niu, Yuanke Zhang, Changwu Li, Zhengfan Zhang, Shuangshuang Guo, Bingying Ding

**Affiliations:** Hubei Key Laboratory of Animal Nutrition and Feed Science, Engineering Research Center of Feed Protein Resources on Agricultural By-Products, Wuhan Polytechnic University, Ministry of Education, Wuhan 430023, China

**Keywords:** vitamin A, necrotic enteritis, antioxidant, intestinal barrier, broiler

## Abstract

**Simple Summary:**

Coccidia and *C. perfringens* are the main accomplices in broiler necrotizing enteritis (NE), and NE causes oxidative stress and intestinal damage in broilers, which will result in huge economic losses to the poultry industry. Vitamin A (VA) is an essential micronutrient of the diet, and the beneficial effects of VA on vision, growth, and development have been intensively investigated. At present, the effects of VA on intestinal barrier and antioxidant functions in broilers have not been systematically reported. The present study was conducted to investigate the effects of dietary supplementation with vitamin A on the antioxidant and intestinal barrier function of broilers co-infected with coccidia and *C. perfringens* (CCP). Our outcomes showed that dietary VA might help a little with intestinal barrier function; nonetheless, it failed to alleviate the negative effects of CCP on the antioxidant function in broilers. Our study has guiding significance for the dose of VA in the diet of broilers, and it might arouse readers’ strong interest.

**Abstract:**

Necrotic enteritis (NE) impairs poultry production and causes great economic loss. The nutritional regulation of diets has the potential to alleviate NE. The present study was conducted to investigate the effects of dietary supplementation with vitamin A (VA) on the antioxidant and intestinal barrier function of broilers co-infected with coccidia and *C. perfringens* (CCP). In a 2 × 2 factorial arrangement, 336 one-day-old Ross 308 broilers were divided into four treatments with two levels of VA (0 or 12,000 IU/kg) and challenged with or without CCP. The animal trial lasted for 42 days. The results showed that dietary supplemental VA improved body weight gain (BWG) and the feed intake (FI), and the FI was negatively affected by CCP. Additionally, the levels of catalase (CAT) in the serum, total superoxide dismutase (T-SOD), and CAT in the jejunum and glutathione peroxidase (GSH-Px) in the liver decreased with the CCP challenge (*p* < 0.05). The mRNA levels of *SOD*, *CAT*, *GSH-Px1*, and *GSH-Px3* in the liver and jejunum were upregulated by the CCP challenge (*p* < 0.05). In addition, the level of serum diamine oxidase (DAO), and the mRNA level of *ZO-1* were also upregulated with the CCP challenge. Dietary supplementation with VA contributed to the intestinal villi height and the mRNA level of *Mucin-2* in the jejunum (*p* < 0.05). Additionally, dietary VA had the ability to alleviate the upregulation of SOD in the liver and *SOD*, *CAT*, *GSH-Px1*, *GSH-Px3*, *ZO-1*, and *claudin-1* in the jejunum with the CCP challenge (*p* < 0.05). However, the mRNA level of *GSH-Px3* and the levels of SOD in the liver and jejunum were downregulated with the VA supplementation in the diet. In conclusion, dietary VA improved the growth performance and the intestinal barrier function; nonetheless, it failed to alleviate the negative effects of CCP on the antioxidant function in broilers.

## 1. Introduction

Some pathogenic microorganisms and stimulators are prone to causing oxidative stress and intestinal damage in poultry production. A large number of reactive oxygen species (ROS) are produced under oxidative stress, which raises the levels of malondialdehyde (MDA) and myeloperoxidase (MPO) in birds and leads to tissue damage [1,2,3]. Additionally, ROS could also induce an inflammatory response by activating nuclear factor κB (NF-κB) [4]. The intestine is the main site for the digestion and absorption of nutrients, and it is also the largest immune organ of the body. A healthy intestine is a material mechanism for the high production of poultry [5]; while oxidative stress and inflammatory responses cause intestinal damage in poultry, an injured intestine opens the door for pathogenic microorganisms and stimulators, which will have negative effects on the growth performance and health of poultry [6]. Coccidia and *C. perfringens* are the main accomplices in necrotic enteritis (NE) in poultry, and NE causes the abnormal expression of antioxidant enzymes and peroxide products [7]. In addition, intestinal barrier-related proteins such as tight-junction proteins might be overexpressed in response to inflammation [8,9], and an excessive inflammatory response downregulates their expression and destroys the intestinal barrier [10]. The oxidative stress and intestinal damage induced by NE has negative effects on animal performance, which results in huge economic losses for the poultry industry.

Vitamin A (VA) is an essential micronutrient of the diet, and its beneficial effects on vision, growth and development have been intensively investigated [11]. In addition, it was demonstrated in a number of studies that VA has the potential to improve the antioxidant and intestinal barrier function. It has been suggested that VA enhances the bioavailability of selenium, which improves the activities of glutathione peroxidase (GSH-Px) [12]. Li also reported that carotenoid has the direct power of antioxidative activities [13]. Another study demonstrated that VA has the ability to maintain the integrity of the epithelium and mucosa [14]. Additionally, some studies suggested that a lack of VA in the diet might provide an opportunity for NE and other intestinal diseases in broilers [11,15]. At present, the effects of VA on NE in broilers have not been fully characterized. Taking the above studies into account, VA might alleviate the adverse effects of NE on broilers via improving antioxidant and barrier function.

We believed it would be very interesting to investigate the effects of VA on NE caused by coccidia and *C. perfringens* in broilers. In the present study, coccidia and *C. perfringens* were used to establish an NE model in broilers to investigate the effects of dietary supplementation with VA on the antioxidant and intestinal barrier function of NE birds.

## 2. Materials and Methods

### 2.1. Experimental Design and Diets

All experimental procedures used were approved by the Animal Care and Use Committee of Wuhan Polytechnic University.

A 2 × 2 factorial design was implemented in which broilers were challenged with or without coccidia and *C. perfringens* (CCP), and their diets were supplemented with or without VA. A total of 336 healthy one-day-old Ross 308 broilers with similar initial body weights were randomly assigned to four groups. There were 7 replicates in each group, and 12 birds (6 males and 6 females) in each replicate. The birds in the control (CTR) and CCP groups were fed a basal diet without supplementation of VA, and the basal diet was formulated accorded to the feeding standard of China (Ministry of Agriculture of China, 2004) and NRC requirements (National Research Council, 1994) (Table 1). The birds in the VA and VA + CCP groups were fed basal diets supplemented with 12,000 IU/kg of VA. Additionally, the birds in the CCP and VA + CCP groups were challenged with coccidia on day 14 of the trial, and treated with 1 mL 1 × 10^8^ CFU/mL C. perfringens from day 18 to day 20 of the trial. The *C. perfringens* (CVCC2030) was purchased from the China Veterinary Microbial Culture Collection and Management Center (Beijing, China), and coccidia from Foshan Zhengdian Biotechnology Co., Ltd., (Foshan, Guangdong, China) was used in the present study. The quadrivalent anti-coccidiosis vaccine consisted of 1 × 10^5^ oocysts of the E. acervuline strain PAHY as well as 5 × 10^4^ oocysts of the E. necatrix strain PNHZ, E. tenella strain PTMZ, and E. maxima strain PMHY. Every bird was recommended to be inoculated with 1100 ± 110 sporulated oocysts according to the instructions, and the dose used in the present study was 20 times that of the recommended. The birds in the CTR and VA groups were treated with an equal volume of saline at the same time. All birds were raised in cages and had free access to feed and water. A 24 h light regime was carried out throughout the animal trial, and the room temperature was controlled at 30–33 °C in the first week, and then it decreased by 2–3 °C in each week until reaching 22 °C, and this temperature was kept till the end of trial. The animal trial lasted for 42 days. The broilers were weighed at days 1, 13, 21, and 42 of the trial, and the body weight gain (BWG), feed intake (FI), and feed conversion ratio (FCR) were measured. On days 28 and 35, 1 male and 1 female broiler in each replicate were selected to harvest the blood from the underwing vein, and then those birds were injected intravenously with 50 mg/kg body weight (BW) sodium pentobarbital and quickly slaughtered for sample collection. The blood was collected and centrifuged at 3000× *g* at 4 °C for 10 min to obtain the serum. The liver and jejunum were collected and frozen in liquid nitrogen, and then they were transferred to a −80 °C freezer for storage.

### 2.2. Antioxidative Enzymes and Peroxidation Products

A total of 0.1 g liver or jejunum was placed in a 1.5 mL centrifuge tube, and 0.9 mL saline was added. Then, the samples were homogenized at 4 °C. The mixture was centrifuged at 3500× *g* at 4 °C for 15 min to collect the supernatants. Commercial assay kits were purchased from Nanjing Jiancheng Institute of Biological Engineering, Nanjing, China. The levels of total antioxidant capacity (T-AOC), catalase (CAT), hydrogen peroxide (H_2_O_2_), glutathione peroxidase (GSH-Px), malondialdehyde (MDA), and total superoxide dismutase (T-SOD) in the serum, liver, and jejunum were measured according to the manufacturer’s instructions.

### 2.3. Serum Diamine Oxidase and Intestinal Morphology

The level of diamine oxidase (DAO) in the serum was determined using commercially available kits from the Nanjing Jiancheng Institute of Biological Engineering, Jiangsu, China. Approximately 1 cm of the duodenum, jejunum, and ileum were collected and placed in a 4% paraformaldehyde solution, and then the intestinal tissue was dehydrated, transparent, waxed, and embedded, after which a slicer was used to cut the tissue to a thickness of 3–5 microns. Finally, the sections were stained with hematoxylin and eosin. A previous study [16] was used to measure the villus height (VH) and crypt depth (CD). Briefly, the intestinal sections of 14 birds (7 males and 7 females) in each treatment were selected, and for each intestinal section, 10 morphologically intact intestinal villi were selected for measurements. These 10 values were averaged and used as the result of this intestinal segment for subsequent analysis. The Olymps BX-41TF microscope was used for observation and a thousand-screen high-definition color pathology graphic analysis system was used to measure the VH and CD. The ratio of the VH to CD (VH/CD) was calculated.

### 2.4. Transcription Level of Genes in the Liver and Jejunum

Approximately 0.1 g of hepatic and jejunal sample was put in a 1.5 mL RNA-free centrifuge tube, and 1 mL Trizol reagent (Invitrogen Life Technologies, Carlsbad, CA, USA) was added. Then, the mixture was homogenized at 4 °C. The total RNA was extracted according to the method described by Hou et al. [17]. The concentration and purity of the total RNA was quantified by measuring its optical density at 260 and 280 nm with the spectrophotometer Nano Drop® ND-2000 UV-VIS (Thermo Scientific, Wilmington, DE, USA). The RNA integrity was verified by agarose gel electrophoresis, and then the total RNA was reverse transcribed to obtain the cDNA followed with the protocol of the gDNA Eraser (Takara Biotechnology (Dalian) Co., Ltd., Dalian, China). A quantitative real-time PCR assay was performed with the 7500-fluorescence detection system (Applied Biosystems, Foster City, CA, USA). The SYBR Premix Ex Taq kit (Takara Biotechnology (Dalian) Co., Ltd., Dalian, China), the cDNA of each sample, and primers were used in its performance. The PCR reaction program was as follows: an initial denaturation step set at 95 °C for 30 s, 40 cycles at 95 °C for 5 s, and an annealing and extension temperature at 60 °C for 34 s. The PCR products from each primer pair were subjected to a melting curve analysis and subsequent agarose gel electrophoresis. The gene expression was quantified using the comparative threshold cycle method [18], and the data are expressed relative to the CTR group. The mRNA levels of the nuclear factor erythroid 2-related factor 2 (*Nrf-2*), superoxide dismutase 1 (*SOD-1*), catalase (*CAT*), glutathione peroxidase 1 (*GSH-px1*), and glutathione peroxidase 3 (*GSH-px3*) in the liver and jejunum were measured. Additionally, the transcription levels of *claudin-1*, *occludin*, zonula occludens-1 (*ZO-1*), and *mucin-2* in the jejunum were detected. *β-Actin* was used as an internal reference gene, and the primer sequences of each gene are described in Table 2.

### 2.5. Statistical Analysis

The general linear model program in SPSS 23.0 software (SPSS Inc., Chicago, IL, USA) was used to analyze the data. Two-way ANOVA was performed in a 2 × 2 factor arrangement to analyze the effects of the CCP challenge and VA supplementation as well as the interaction of these two factors. When interactive effects were observed, one-way ANOVA and Tukey’s multiple comparisons were used. *p* < 0.05 was considered to be significant. GraphPad Prism 8.0 software (GraphPad software, LLC, San Diego, CA, USA) was used to graph the data.

## 3. Results

### 3.1. Growth Performance

A dietary supplemental of 12,000 IU/kg VA was also able to elevate the BWG and FI from day 14 to day 20 of the trial (*p* < 0.05) (Table 3), and the FI from day 21 to day 42 of the trial decreased with the coccidia and *C. perfringens* challenge.

### 3.2. Antioxidative Enzymes and Peroxidation Products

The activity of CAT in the serum decreased on day 35 with the coccidia and C. perfringens challenge (*p* < 0.05) (Table 4). Dietary supplementation with 12,000 IU/kg VA raised the level of MDA on day 28 and decreased the level of H_2_O_2_ on day 35 in the serum (*p* < 0.05). In the liver, the levels of T-AOC and MDA increased, and the activity of GSH-Px decreased on day 28 with the coccidia and C. perfringens challenge (*p* < 0.05) (Table 5). Dietary VA failed to alleviate these indices, and the T-AOC level and T-SOD activity in the liver dropped on day 35 with VA supplementation in the diet (*p* < 0.05). Dietary VA might have a negative effect on the antioxidant function of broilers, a phenomenon that was also found in the jejunum. The T-AOC level and T-SOD activity on day 28 and the activities of CAT and T-SOD on day 35 decreased with VA addition (*p* < 0.05) (Table 6). However, dietary VA decreased the contents of H_2_O_2_ and MDA on day 35 (*p* < 0.05). The T-SOD activity on day 28 as well as the T-SOD and CAT activities on day 35 decreased with the coccidia and *C. perfringens* challenge (*p* < 0.05).

### 3.3. The mRNA Levels of Antioxidant-Related Genes

Dietary supplementation with VA downregulated the mRNA levels of *GSH-Px1* and *GSH-Px3* in the liver on days 28 and 35 (*p* < 0.05) (Table 7). The mRNA levels of *SOD* and *CAT* in the liver on day 35 were upregulated by the CCP challenge (*p* < 0.05). Dietary VA could alleviate the upregulation of SOD in the liver in the CCP-challenged broilers. The mRNA levels of *Nrf-2*, *SOD*, *CAT*, *GSH-Px1*, and *GSH-Px3* in the jejunum on day 35 were upregulated by the CCP challenge (*p* < 0.05) (Table 8). Dietary VA was able to alleviate the upregulation of *SOD*, *CAT*, *GSH-Px1*, and *GSH-Px3* in the CCP-challenged broilers (*p* < 0.05). The mRNA level of *CAT* in the jejunum on day 35 was upregulated by VA addition in the diet (*p* < 0.05), whereas the transcript level of *GSH-Px3* was downregulated with the VA supplementation (*p* < 0.05).

### 3.4. Intestinal Barrier Function

**Figure 1:**
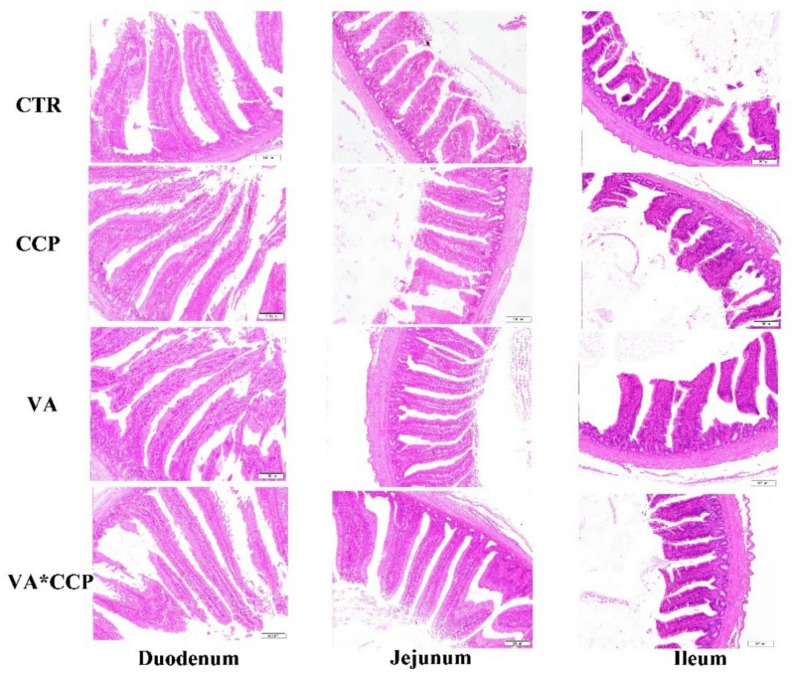
The intestinal morphological parameters on day 28. CTR = control group; CCP = co-infected with coccidia and *C. perfringens* group; VA = dietary supplementation with 12,000 IU/kg VA group; VA + CCP = dietary supplementation with 12,000 IU/kg VA and co-infected with coccidia and *C. perfringens* group. Each image has a 200 μm scale, and the image is magnified 100 times.

**Figure 2:**
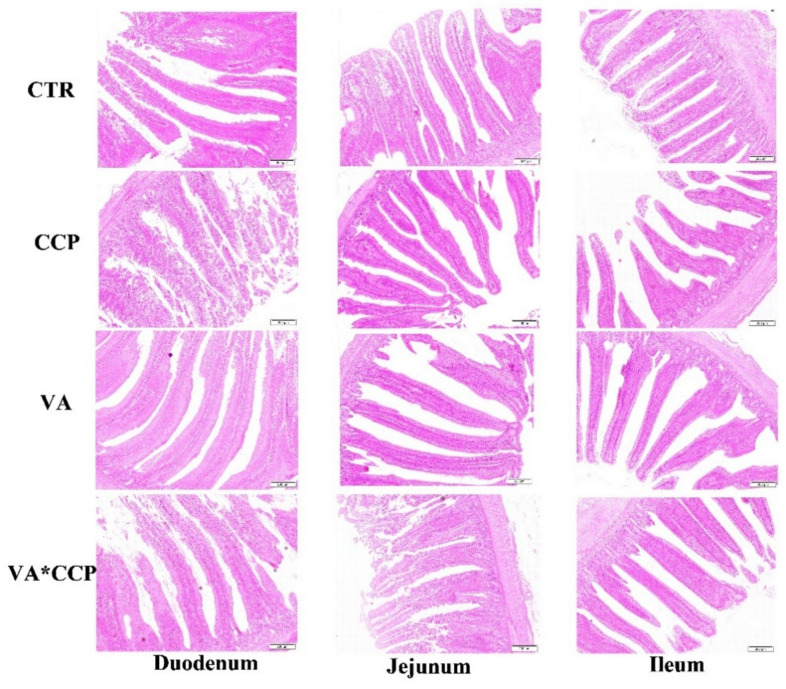
The intestinal morphological parameters on day 35. CTR = control group; CCP = co-infected with coccidia and *C. perfringens* group; VA = dietary supplementation with 12,000 IU/kg VA group; VA* CCP = VA × CCP = dietary supplementation with 12,000 IU/kg VA and co-infected with coccidia and *C. perfringens* group. Each image has a 200 μm scale, and the image is magnified 100 times.

**Figure 3:**
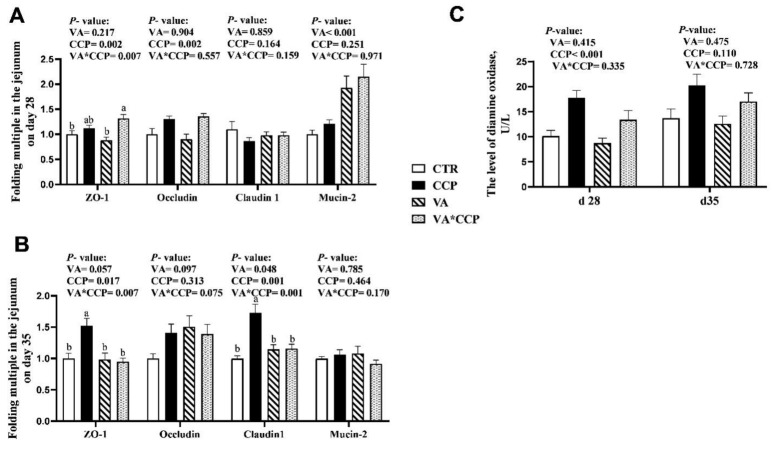
Transcription levels of jejunal barrier-related genes and serum DAO levels: (**A**,**B**) results of the jejunum barrier genes on days 28 and 35, respectively; (**C**) results of serum DAO. ^a, b^ Different pillar without common superscripts differ significantly (*p* < 0.05). Data are presented as the mean ± standard deviation. CTR = control group; CCP = co-infected with coccidia and *C. perfringens* group; VA = dietary supplementation with 12,000 IU/kg VA group; VA + CCP = dietary supplementation with 12,000 IU/kg VA and co-infected with coccidia and *C. perfringens* group.

The statistical parameters of the intestinal tract were not significantly affected by the CCP challenge; however, the surface of the intestinal tract showed roughness, and the intestinal tract was scattered and varied in shape and size (*p* > 0.05) (Table 9 and Table 10) (Figure 1 and Figure 2). Dietary supplementation with VA was able to raise the villi height (VH) of the duodenum, jejunum, and ileum on day 28 (Table 9) and the VH of ileum on day 35 (*p* < 0.05) (Table 10). In the VA treated, the intestinal tract was uniform in size and neat in direction. Fluff indicated smoothness (Figure 1 and Figure 2). Co-infection with CCP elevated the mRNA levels of *occludin* and *ZO-1* in the jejunum on day 28. Additionally, the transcript levels of *ZO-1* and *claudin-1* were also upregulated on day 35 (*p* < 0.05) (Figure 3A,B). Interestingly, dietary VA could alleviate the upregulation of *ZO-1* and *claudin-1* on day 35, and the mRNA level of *mucin-2* was upregulated on day 28 with VA supplementation in the diet. In addition, the level of DAO in the serum on day 28 was increased by the CCP challenge (*p* < 0.05) (Figure 3C). Dietary VA also failed to alleviate this index.

## 4. Discussion

NE is a multifactorial inflammatory disease of the intestine, and *C. perfringens* is one of the main causes [19], whereas with *C. perfringens* it only occurs in concert with other factors that cause intestinal damage, for example, a coccidia infection and the wheat basal diet [20,21]. In the present study, *C. perfringens* and coccidia were used to establish a model of NE. The antioxidant function of NE-infected broilers was weakened, and the intestinal barrier function was impaired [22,23]. The antioxidant system and the pro-oxidative system were maintained at a stable level, and some adverse factors, such as pathogens, viruses, and other stressors, could disturb this balance. At the same time, the body will have an oxidative stress response. Accordingly, some peroxidation products, such as ROS, malondialdehyde (MDA), and myeloperoxidase (MPO), will be increased [24]. In this study, the levels of MDA in the liver and jejunum were increased by the CCP challenge, and it was only observed for a short time after infection. Antioxidant enzymes, such as T-SOD, CAT, and GSH-Px, have the ability to mitigate the elevated peroxidation products [25]. The total antioxidant capacity (T-AOC) is regarded as the ability to maintain the balance of the antioxidant and peroxidation systems [26]. In the present study, co-infection with coccidia and *C. perfringens* decreased the activities of CAT, T-SOD, and GSH-Px. These results demonstrate that the combined infection of coccidia and *C. perfringens* caused oxidative stress. This is basically consistent with previous studies [7]. We observed that dietary supplementation with 12,000 IU/kg VA failed to alleviate the oxidative stress of NE-infected broilers. Moreover, the activities of T-SOD and GSH-Px in the liver decreased with VA supplementation in the diet. This study suggests that the activities of SOD, CAT, and GSH-Px significantly increased with 10 mg/kg β-carotene, which is a VA precursor. Conversely, 20 mg/kg of dietary β-carotene decreased the activities of SOD and GSH-Px [27]. The dual effects of VA on antioxidant function may be related to its dose. This illuminated for us that a dose of 12,000 IU/kg VA might be detrimental to the antioxidant function of broilers, and it was worthy of further study.

The Kelch-like ECH-associated protein 1 (Keap-1)-Nrf-2 system is a major regulatory pathway against oxidative stress. Keap-1 and Nrf-2 are tightly tied together under normal conditions, and they are disassembled with stressor challenge. Then, Nrf-2 is translocated into the nucleus to initiate the transcription of antioxidant genes, such as *SOD*, *CAT*, and *GSH-Px* [28]. In the present study, we observed that the mRNA levels of *Nrf-2*, *SOD*, *CAT*, *GSH-Px1*, and *GSH-Px3* in the jejunum were upregulated by the CCP challenge. Additionally, the transcript levels of *SOD* and *CAT* in the liver were also upregulated. Our outcomes suggest that the antioxidant system of broilers was activated to respond to the infection by CCP. In addition, dietary VA was able to alleviate the upregulation of antioxidant enzyme genes with CCP challenge. However, the transcript level of *GSH-Px3* was downregulated with VA supplementation in the diet. Although VA was helpful to the antioxidant capacity of the NE-infected broilers at the gene expression level, the outcomes of the antioxidant enzymes and their products suggest that a lower dose of VA than 12,000 IU/kg should be recommended. It is worth mentioning that these changes in antioxidant enzyme genes were not significant for a short period of time after the infection. Interestingly, it was significant for 2 weeks after the infection. A study suggested that the growth performance of broilers infected with NE was negatively affected, and it was only observed for a short period of time after infection [10]. A mechanism of self-compensating growth and homeostasis maintenance may be activated after infection [29].

Diamine oxidase (DAO) is an intracellular enzyme that catalyzes diamine in the mucosal or ciliated epithelial cells of the mammalian small intestine. It contributes to protecting the intestinal mucosa via promoting cell repair [30]. An early enteral nutrition study demonstrated that the concentration of DAO in plasma was raised sharply when the intestinal mucosa was damaged [31]. In the present study, the level of DAO in the plasma was elevated with the CCP challenge. A study suggested that in the intestinal damage model, the DAO level in the intestine was highly negatively correlated with it in the serum [17]. This evidence indicates that a combined infection with CCP caused intestinal damage in broilers. An intact intestinal structure is the basis for the intestinal tract to exert some physiological functions. The degree of development of the intestinal morphology could be evaluated by the indices of VH, CD, and VH/CD [32]. In our study, the surface of the intestinal tract challenged with CCP showed roughness, and the intestinal tract was scattered and varied in shape and size. However, the levels of VH, CD, and VH/CD were not affected by the CCP challenge. This is inconsistent with our expectations. To explain the observed phenomenon, we might consider that a wheat basal diet should be required in the establishment of an NE model using CCP infections. This might make the intestinal damage observation more significant. Intriguingly, dietary supplementation with 12,000 IU/kg VA contributed to the VH of intestine. A study also implicated that dietary β-carotene raised the intestinal VH [33]. This might be one of the reasons why VA was useful for the growth performance of broilers in the present study.

Tight junction proteins between intestinal cells help maintain the intestinal barrier function. ZO-1, claudin-1, and occludin play key roles in regulating the barrier function of intestinal epithelium [34]. Some studies demonstrated that the transcriptional levels of genes encoding tight junction proteins are upregulated under inflammatory conditions [8,9]. In the present study, co-infection with CCP also upregulated the mRNA levels of *occludin*, *ZO-1*, and *claudin-1* in the intestine. A possible explanation is that the inflammation activated the intestinal self-protection mechanism, and the high expression of tight junctions was used to protect the intestine from pathogen stimulation. Interestingly, dietary supplementation with 12,000 IU/kg VA could alleviate the upregulation of *ZO-1* and *claudin-1* with CCP challenge. In addition, the mRNA level of *mucin-2* was upregulated with VA addition, which is consistent with another study [15]. Mucin-2 secreted by goblet cells adheres to the surface of the intestinal villi and is considered the first physical barrier of the intestine [35]. Symbiotic bacteria in the intestinal tract generally colonize the outer layer of the intestinal mucosa. Some probiotics stimulate goblet cells to secrete mucin, which strengthens the mucin layer to protect the intestinal tract from an invasion of pathogenic bacteria [36]. In the present study, dietary supplemental VA might be useful for the proliferation of some probiotics and the proliferation and differentiation of intestinal goblet cells of broilers. Further research is needed to investigate this issue. Taking the beneficial effects of VA on intestinal VH and the mRNA levels of tight junction proteins into account, dietary supplementation with 12,000 IU/kg VA might offer exciting opportunities for the intestinal barrier function of NE-infected broilers.

## 5. Conclusions

Dietary supplementation with 12,000 IU/kg VA might have a negative effect on the antioxidant function of broilers. However, it had great potential to alleviate the adverse effect of co-infection with CCP on intestinal barrier function and promoted intestinal development.

## Figures and Tables

**Table 1:** The feed ingredient composition and nutrient levels, on an air-dried basis.

Items (%, Unless Otherwise Indicated)	Starter Period (Days 0 to 21)	Grower Period (Days 22 to 42)
Ingredient		
Corn	51.59	57.68
Soybean meal	40.78	35.15
Soybean oil	3.44	3.66
Dicalcium phosphate	1.92	1.33
Limestone	1.16	1.26
Sodium chloride	0.35	0.35
DL-methionine	0.26	0.13
Choline chloride	0.25	0.20
Trance mineral premix ^1^	0.20	0.20
Vitamin premix ^2^	0.05	0.04
Calculated nutrient levels		
Crude protein	21.50	19.50
ME (MJ/kg)	12.22	12.56
Ca	1.00	1.00
Available P	0.45	0.35
Lysine	1.17	1.04
Methionine + cystine	0.90	0.72

^1^ The trace mineral premix supplied the following per kilogram of diet: copper, 8 mg; iron, 80 mg; zinc, 75 mg; manganese, 100 mg; selenium, 0.15 mg; iodine, 0.35 mg. ^2^ The vitamin premix supplied the following per kilogram of diet: vitamin D, 32,500 IU; vitamin E, 30 IU; vitamin K, 32.65 mg; vitamin B1, 2 mg; vitamin B2, 6 mg; vitamin B12, 0.025 mg; biotin, 0.0325 mg; folic acid, 1.25 mg; pantothenic acid, 12 mg; nicotinic acid, 50 mg.

**Table 2:** The primer sequences list ^1^.

Gene Name		Primer Sequence (5′ to 3′)	NCBI Number
*β-actin*	Forward	ACTCTGGTGATGGTGTTAC	NM 205518
	Reverse	GGCTGTGATCTCCTTCTG	
*Nrf-2*	Forward	ATCACCTCTTCTGCACCGAA	NM 205117
	Reverse	GCTTTCTCCCGCTCTTTCTG	
*SOD-1*	Forward	GGTGCTCACTTTAATCCTG	NM 205064
	Reverse	CTACTTCTGCCACTCCTCC	
*CAT*	Forward	GGTTCGGTGGGGTTGTCTTT	NM_001031215.2
	Reverse	CACCAGTGGTCAAGGCATCT	
*GSH-* *Px1*	Forward	GACCAACCCGCAGTACATCA	NM_001277853.2
	Reverse	GAGGTGCGGGCTTTCCTTTA	
*GSH-* *Px3*	Forward	AAGTGCCAGGTGAACGGGAAGG	NM 001163232
	Reverse	AGGGCTGTAGCGGCGGAAAG	
*Claudin-1*	Forward	CATACTCCTGGGTCTGGTTGGT	AY750897
	Reverse	GACAGCCATCCGCATCTTCT	
*Occludin*	Forward	ACGGCAGCACCTACCTCAA	D21837.1
	Reverse	GGGCGAAGAAGCAGATGAG	
*ZO-1*	Forward	CTTCAGGTGTTTCTCTTCCTCCTC	XM_413773
	Reverse	CTGTGGTTTCATGGCTGGATC	
*Mucin-2*	Forward	TTCATGATGCCTGCTCTTGTG	XM_421035
	Reverse	CCTGAGCCTTGGTACATTCTTGT	

^1^ The primers were designed and synthesized by Shanghai Sangon Bioengineering Co., Ltd., Shanghai, China. *Nrf-2* = nuclear factor erythroid 2-related factor 2; *SOD-1* = superoxide dismutase 1; *CAT* = catalase; *GSH-Px1* and GSH-Px3 = glutathione peroxidase 1 and 3. *Claudin-1*, *occludin*, and *ZO-1* were tight junction proteins.

**Table 3:** The growth performance of broilers after the challenge (days 14 to 42).

Item		Days 14 to 20			Days 21 to 42		
		BWG (g)	FI (g)	FCR	BWG (g)	FI (g)	FCR
CTR		176.2	302.3	1.755	1307.6	1966.4	1.506
CCP		236.4	368.6	1.558	1391.8	2125.2	1.529
VA		186.9	289.1	1.582	1306	1878.3	1.444
VA × CCP		240.1	432.6	1.798	1388.3	2019.1	1.456
SEM		7.20	16.60	0.07	76.00	32.50	0.02
Main effect	VA (−)	181.2 ^b^	296.2 ^b^	1.675	1306.8	1925.7	1.478
	VA (+)	238.2 ^a^	400.6 ^a^	1.678	1390	2072.1	1.492
	CCP (−)	206.3	335.5	1.656	1349.7	2045.8 ^a^	1.517
	CCP (+)	215.6	366.4	1.698	1350.3	1954.1 ^b^	1.45
*p*-Value	VA	0.008	0.001	0.947	0.798	0.531	0.741
	CCP	0.481	0.337	0.818	0.542	0.020	0.193
	VA × CCP	0.704	0.150	0.167	0.856	0.988	0.912

^a, b^ Means in the same row without common superscripts differ significantly (*p* < 0.05). CTR = control group; CCP = co-infected with coccidia and *C. perfringens* group; VA = dietary supplementation with 12,000 IU/kg VA group; VA × CCP = dietary supplementation with 12,000 IU/kg VA and co-infected with coccidia and *C. perfringens* group; BWG = body weight gain; FI = feed intake; FCR = feed conversion ratio; SEM = standard error of the mean.

**Table 4:** Effect of vitamin A on the levels of antioxidant enzymes and peroxidation products in the serum of CCP-challenged broilers.

Item		d 28					d 35				
		CAT	H_2_O_2_	MDA	GSH-px	T-SOD	CAT	H_2_O_2_	MDA	GSH-px	T-SOD
		U/mg Prot	nmol/mg Prot		U/mg Prot		U/mg Prot	nmol/mg Prot		U/mg Prot	
CTR		1.30	40.94	2.13	1493.92	90.57	1.17	24.47	2.76	1543.70	101.59
CCP		1.10	34.23	2.17	1398.13	98.83	0.67	23.00	3.18	1641.23	102.87
VA		0.78	37.48	2.72	1556.61	96.03	1.07	17.63	3.12	1544.98	105.19
VA × CCP		0.77	38.29	2.76	1461.46	108.17	0.39	19.03	3.30	1493.25	103.79
SEM		0.69	12.12	0.70	208.28	13.79	0.58	5.06	0.54	361.49	11.19
Main effect	VA (−)	1.20	37.59	2.15 ^a^	1446.02	94.70	0.92	23.74 ^a^	2.97	1592.47	102.23
	VA (+)	0.77	37.88	2.74 ^b^	1509.03	102.10	0.73	18.33 ^b^	3.21	1519.11	104.49
	CCP (−)	1.04	39.21	2.42	1525.26	93.30 ^b^	1.12 ^a^	21.05	2.94	1544.34	103.39
	CCP (+)	0.93	36.26	2.47	1429.79	103.50 ^a^	0.53 ^b^	21.02	3.24	1567.24	103.33
*p*-Value	VA	0.090	0.928	0.018	0.345	0.073	0.286	<0.001	0.158	0.537	0.541
	CCP	0.671	0.371	0.855	0.156	0.015	0.003	0.981	0.083	0.847	0.987
	VA × CCP	0.700	0.255	0.989	0.996	0.631	0.607	0.306	0.478	0.530	0.715

^a, b^ Means in the same row without common superscripts differ significantly (*p* < 0.05). CTR = control group; CCP = co-infected with coccidia and *C. perfringens* group; VA = dietary supplementation with 12,000 IU/kg VA group; VA × CCP = dietary supplementation with 12,000 IU/kg VA and co-infected with coccidia and *C. perfringens* group; Prot = protein; SEM = standard error of the mean.

**Table 5:** Effect of vitamin A on the levels of antioxidant enzymes and peroxidation products in the liver of CCP-challenged broilers.

Item		d 28						d 35					
		T-AOC	CAT	H_2_O_2_	MDA	GSH-px	T-SOD	T-AOC	CAT	H_2_O_2_	MDA	GSH-px	T-SOD
		mM	U/mg Prot	nmol/mg Prot		U/mg Prot		mM	U/mg Prot	nmol/mg Prot		U/mg Prot	
CTR		1.18 ^b^	5.69	33.55	1.01	187.43	1187.10	1.08	10.48	48.40	1.11	189.97	1284.11 ^b^
CCP		1.43 ^a^	5.43	31.03	1.29	147.73	1223.18	1.04	10.11	43.97	1.03	187.12	1452.47 ^a^
VA		1.41 ^a^	5.06	44.77	1.00	145.66	1210.10	0.95	10.50	46.48	1.25	219.30	1123.94 ^c^
VA × CCP		1.44 ^a^	4.71	41.56	1.10	115.65	1195.11	0.90	7.97	42.56	1.16	216.90	1088.80 ^c^
SEM		0.14	1.76	7.86	0.30	41.83	105.34	0.16	2.67	12.39	0.27	35.79	198.04
Main effect	VA (−)	1.30 ^b^	5.56	32.29 ^b^	1.15	167.58 ^a^	1205.14	1.06 ^a^	10.29	46.18	1.07	188.55 ^b^	1368.29 ^a^
	VA (+)	1.43 ^a^	4.88	43.16 ^a^	1.05	130.65 ^b^	1202.61	0.93 ^b^	9.24	44.52	1.20	218.10 ^a^	1106.37 ^b^
	CCP (−)	1.30 ^b^	5.37	39.16	1.01 ^b^	166.54 ^a^	1198.60	1.01	10.49	47.44	1.18	204.64	1204.03
	CCP (+)	1.43 ^a^	5.07	36.30	1.20 ^a^	131.69 ^b^	1209.15	0.97	9.04	43.26	1.09	202.01	1270.63
*p*-Value	VA	<0.001	0.161	<0.001	0.173	<0.001	0.930	0.007	0.216	0.670	0.111	0.007	<0.001
	CCP	<0.001	0.525	0.061	0.017	0.001	0.715	0.327	0.092	0.289	0.271	0.801	0.127
	VA × CCP	<0.001	0.931	0.817	0.261	0.611	0.378	0.880	0.206	0.948	0.935	0.983	0.022

^a, b, c^ Means in the same row without common superscripts differ significantly (*p* < 0.05). CTR = control group; CCP = co-infected with coccidia and *C. perfringens* group; VA = dietary supplementation with 12,000 IU/kg VA group; VA × CCP = dietary supplementation with 12,000 IU/kg VA and co-infected with coccidia and *C. perfringens* group; Prot = protein; SEM = standard error of the mean.

**Table 6:** Effect of vitamin A on the levels of antioxidant enzymes and peroxidation products in the jejunum of CCP-challenged broilers.

Item		d 28						d 35					
		T-AOC	CAT	H_2_O_2_	MDA	GSH-px	T-SOD	T-AOC	CAT	H_2_O_2_	MDA	GSH-px	T-SOD
		mM	U/mg Prot	nmol/mg Prot		U/mg Prot		mM	U/mg Prot	nmol/mg Prot		U/mg Prot	
CTR		0.87	1.62	4.74 ^a^	1.92 ^b^	41.60	1274.41	0.45	1.51	3.89	0.68	21.32	1166.59
CCP		0.86	0.98	2.93 ^b^	11.31 ^a^	38.65	997.07	0.53	1.35	4.86	0.52	21.08	789.92
VA		0.38	1.25	4.42 ^a^	2.52 ^b^	24.80	953.01	0.53	1.31	3.24	0.38	18.05	849.46
VA × CCP		0.37	1.28	4.54 ^a^	1.05 ^b^	26.71	779.95	0.57	0.63	3.34	0.45	20.79	555.99
SEM		0.26	0.85	1.37	4.78	11.54	323.98	0.10	0.71	1.21	0.29	8.61	313.54
Main effect	VA (−)	0.86 ^a^	1.30	3.83 ^b^	6.61 ^a^	40.12 ^a^	1135.74 ^a^	0.49 ^b^	1.43 ^a^	4.37 ^a^	0.60 ^a^	21.20	978.25 ^a^
	VA (+)	0.38 ^b^	1.26	4.48 ^a^	1.79 ^b^	25.75 ^b^	866.48 ^b^	0.55 ^a^	0.97 ^b^	3.29 ^b^	0.42 ^b^	19.42	702.72 ^b^
	CCP (−)	0.62	1.44	4.58 ^a^	2.22 ^b^	33.20	1113.71 ^a^	0.49 ^b^	1.41 ^a^	3.57	0.53	19.68	1008.02 ^a^
	CCP (+)	0.61	1.13	3.73 ^b^	6.18 ^a^	32.68	888.51 ^b^	0.55 ^a^	0.99 ^b^	4.10	0.49	20.94	672.95 ^b^
*p*-Value	VA	<0.001	0.876	0.049	<0.001	<0.001	0.001	0.012	0.010	<0.001	0.020	0.461	<0.001
	CCP	0.696	0.187	0.011	<0.001	0.841	0.004	0.017	0.020	0.065	0.534	0.602	<0.001
	VA × CCP	0.887	0.153	0.004	<0.001	0.346	0.492	0.548	0.140	0.129	0.119	0.535	0.501

^a, b^ Means in the same row without common superscripts differ significantly (*p* < 0.05). CTR = control group; CCP = co-infected with coccidia and *C. perfringens* group; VA = dietary supplementation with 12,000 IU/kg VA group; VA × CCP = dietary supplementation with 12,000 IU/kg VA and co-infected with coccidia and *C. perfringens* group; Prot = protein; SEM = standard error of the mean.

**Table 7:** Effect of vitamin A on mRNA levels of antioxidant-related genes in the liver of CCP-challenged broilers.

Item		d 28					d 35				
		Nrf-2	SOD1	CAT	GSH-px1	GSH-px3	Nrf-2	SOD1	CAT	GSH-px1	GSH-px3
CTR		1.03	1.03	1.04	1.02 ^b^	1.06 ^a^	1.12	1.05 ^b^	1.04	1.01	1.05
CCP		1.39	1.20	1.11	1.00 ^b^	1.01 ^a^	0.90	1.61 ^a^	1.75	1.33	0.80
VA		1.62	1.19	1.33	0.57 ^c^	0.42 ^b^	1.11	0.81 ^b^	0.99	0.68	0.82
VA × CCP		1.62	1.40	1.34	1.47 ^a^	1.33 ^a^	1.32	0.91 ^b^	1.30	0.81	0.63
SEM		0.50	0.41	0.43	0.46	0.61	0.51	0.41	0.58	0.36	0.29
Main effect	VA (−)	1.21 ^b^	1.11	1.07	1.01	1.04	1.01	1.33 ^a^	1.40	1.17 ^a^	0.92 ^a^
	VA (+)	1.62 ^a^	1.30	1.34	1.02	0.88	1.22	0.86 ^b^	1.14	0.75 ^b^	0.72 ^b^
	CCP (−)	1.33	1.11	1.18	0.79 ^b^	0.74 ^b^	1.12	0.93 ^b^	1.02 ^b^	0.85 ^b^	0.93 ^a^
	CCP (+)	1.51	1.30	1.23	1.23 ^a^	1.17 ^a^	1.11	1.26 ^a^	1.53 ^a^	1.07 ^a^	0.71 ^b^
*p*-Value	VA	0.008	0.156	0.054	0.917	0.350	0.242	<0.001	0.120	<0.001	0.045
	CCP	0.225	0.142	0.738	<0.001	0.018	0.968	0.002	0.003	0.038	0.026
	VA × CCP	0.228	0.878	0.842	<0.001	0.009	0.228	0.022	0.225	0.338	0.753

^a, b, c^ Means in the same row without common superscripts differ significantly (*p* < 0.05). CTR = control group; CCP = co-infected with coccidia and *C. perfringens* group; VA = dietary supplementation with 12,000 IU/kg VA group; VA × CCP = dietary supplementation with 12,000 IU/kg VA and co-infected with coccidia and *C. perfringens* group; SEM = standard error of the mean.

**Table 8:** Effect of vitamin A on mRNA levels of antioxidant-related genes in the jejunum of CCP-challenged broilers.

Item		d 28					d 35				
		Nrf-2	SOD1	CAT	GSH-px1	GSH-px3	Nrf-2	SOD1	CAT	GSH-px1	GSH-px3
CTR		1.07	1.02	1.03	1.01 ^b^	1.00	1.09	1.03 ^b^	1.04 ^c^	1.04 ^b^	1.06 ^b^
CCP		0.89	1.29	0.91	0.90 ^b^	0.99	2.19	1.42 ^a^	1.76 ^a^	2.14 ^a^	1.72 ^a^
VA		0.51	0.91	0.76	1.00 ^b^	1.44	1.03	1.04 ^bc^	1.84 ^ab^	1.36 ^b^	0.69 ^bc^
VA × CCP		0.65	1.15	0.87	1.40 ^a^	1.46	1.22	0.84 ^c^	1.30 ^bc^	1.33 ^b^	0.60 ^c^
SEM		0.43	0.34	0.28	0.34	0.50	0.89	0.36	0.55	0.62	0.63
Main effect	VA (−)	0.98 ^a^	1.15	0.97	0.95 ^b^	0.99 ^b^	1.64	1.22 ^a^	1.40	1.59	1.39 ^a^
	VA (+)	0.58 ^b^	1.03	0.81	1.20 ^a^	1.45 ^a^	1.13	0.94 ^b^	1.57	1.35	0.64 ^b^
	CCP (−)	0.79	0.97 ^b^	0.89	1.00	1.22	1.06 ^b^	1.03	1.44	1.20 ^a^	0.87
	CCP (+)	0.77	1.22 ^a^	0.89	1.15	1.22	1.70 ^a^	1.13	1.53	1.74 ^b^	1.16
*p*-Value	VA	0.004	0.246	0.094	0.014	0.003	0.053	0.004	0.241	0.128	<0.001
	CCP	0.852	0.02	0.956	0.134	0.987	0.016	0.327	0.563	0.002	0.058
	VA × CCP	0.212	0.855	0.219	0.010	0.892	0.08	0.004	<0.001	0.001	0.016

^a, b, c^ Means in the same row without common superscripts differ significantly (*p* < 0.05). CTR = control group; CCP = co-infected with coccidia and *C. perfringens* group; VA = dietary supplementation with 12,000 IU/kg VA group; VA × CCP = dietary supplementation with 12,000 IU/kg VA and co-infected with coccidia and *C. perfringens* group; SEM = standard error of the mean.

**Table 9:** Effect of vitamin A on the intestinal morphology of broiler chickens co-infected with CCP on day 28.

Item		Duodenum			Jejunum			Ileum		
		VH (μm)	CD (μm)	VH/CD	VH (μm)	CD (μm)	VH/CD	VH (μm)	CD (μm)	VH/CD
CTR		864.02	93.49	9.57	490.22	64.89	7.65	274.11	60.23	4.55
CCP		930.27	101.32	9.89	527.62	69.75	7.64	303.19	74.37	4.40
VA		969.98	93.74	10.44	596.62	83.54	7.95	406.83	66.43	6.00
VA × CCP		974.72	94.76	10.18	648.38	92.93	7.69	345.28	67.63	5.11
SEM		101.39	16.56	1.66	135.07	32.05	1.81	146.08	21.27	1.51
Main effect	VA (−)	897.14 ^b^	97.40	9.73	508.92 ^b^	67.32 ^b^	7.64	288.65 ^b^	67.30	4.47 ^b^
	VA (+)	972.35 ^a^	94.25	10.31	622.50 ^a^	88.24 ^a^	7.82	376.05 ^a^	67.03	5.55 ^a^
	CCP (−)	917.00	93.61	10.00	543.42	74.22	7.80	340.47	63.33	5.27
	CCP (+)	952.49	98.04	10.03	588.00	81.34	7.66	324.23	71.00	4.75
*p*-Value	VA	0.017	0.505	0.220	0.002	0.019	0.727	0.037	0.965	0.011
	CCP	0.243	0.350	0.951	0.203	0.412	0.794	0.692	0.216	0.204
	VA × CCP	0.311	0.472	0.545	0.836	0.793	0.807	0.271	0.296	0.368

^a, b^ Means in the same row without common superscripts differ significantly (*p* < 0.05). CTR = control group; CCP = co-infected with coccidia and *C. perfringens* group; VA = dietary supplementation with 12,000 IU/kg VA group; VA × CCP = dietary supplementation with 12,000 IU/kg VA and co-infected with coccidia and *C. perfringens* group; SEM = standard error of the mean.

**Table 10:** Effect of vitamin A on intestinal morphology of broiler chickens co-infected with CCP on day 35.

Item		Duodenum			Jejunum			Ileum		
		VH (μm)	CD (μm)	VH/CD	VH (μm)	CD (μm)	VH/CD	VH (μm)	CD (μm)	VH/CD
CTR		819.86	103.85	8.21	719.49	88.15	8.58	519.11	96.53	5.45
CCP		846.28	103.35	8.47	791.60	91.35	9.49	568.82	94.48	6.09
VA		902.61	96.79	9.78	713.61	90.07	8.13	762.75	96.45	8.38
VA × CCP		818.88	93.92	9.18	686.29	83.19	8.52	706.42	109.51	6.51
SEM		141.00	22.22	2.32	110.45	15.23	1.45	328.89	18.78	3.57
Main effect	VA (−)	833.07	103.60	8.34	755.55	89.75	9.04	543.97 ^b^	95.51	5.77
	VA (+)	860.74	95.36	9.48	699.95	86.63	8.33	734.58 ^a^	102.98	7.44
	CCP (−)	861.23	100.32	9.00	716.55	89.11	8.36	640.93	96.49	6.91
	CCP (+)	832.58	98.63	8.83	738.95	87.27	9.01	637.62	102.00	6.30
*p*-Value	VA	0.499	0.211	0.094	0.112	0.477	0.080	0.048	0.165	0.106
	CCP	0.484	0.796	0.796	0.516	0.676	0.108	0.972	0.304	0.550
	VA × CCP	0.182	0.856	0.517	0.154	0.253	0.523	0.574	0.161	0.222

^a, b^ Means in the same row without common superscripts differ significantly (*p* < 0.05). CTR = control group; CCP = co-infected with coccidia and *C. perfringens* group; VA = dietary supplementation with 12,000 IU/kg VA group; VA × CCP = dietary supplementation with 12,000 IU/kg VA and co-infected with coccidia and *C. perfringens* group; SEM = standard error of the mean.

## Data Availability

The datasets produced and/or analyzed during the current study are available from the corresponding author upon reasonable request.
